# Supplementary material for: Atomic high-spin cobalt(II) center for highly selective electrochemical CO reduction to CH3OH
Source: Nat Commun. 2023 Oct 17;14:6550. doi: 10.1038/s41467-023-42307-1 (PMC10582074; doi:10.1038/s41467-023-42307-1)
Supplement: Supplementary file 1 — Supplementary Information [file 41467_2023_42307_MOESM1_ESM.pdf]

Supplementary Information

Atomic High-Spin Cobalt(II) Center for Highly Selective Electrochemical CO Reduction to CH_3OH

Ding et al.

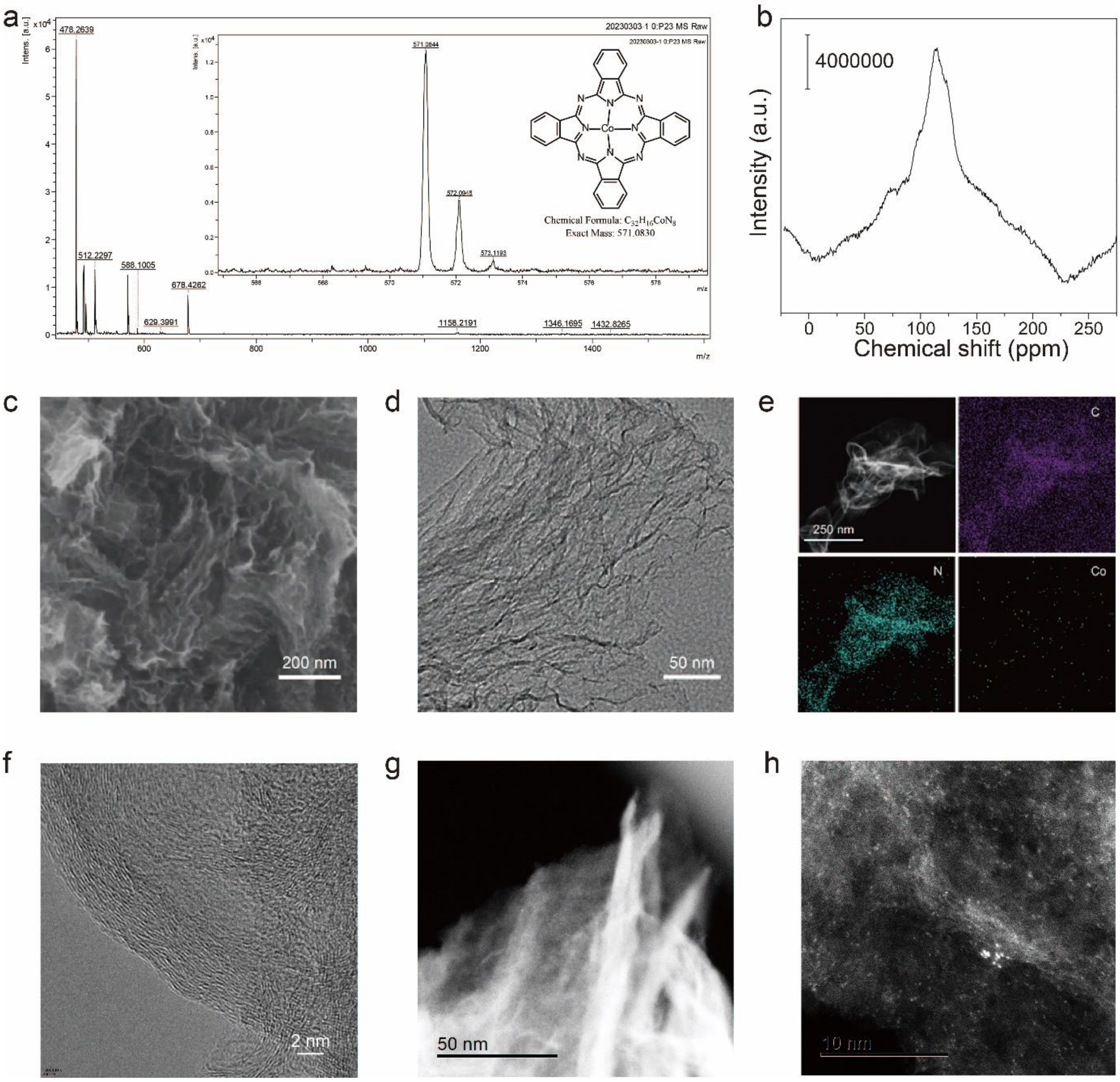

Figure S1 Morphology and structure characterizations for M-CoPc and M-CoPc-400. a, HR-MS and b, ^{13}C NMR spectra for M-CoPc. c, Scanning electron microscopy (SEM) image. d, Transmission electron microscopy (TEM) image. e, Energy dispersive X-ray (EDX) elemental mapping images. f, High-resolution TEM (HR-TEM) image. g, Scanning transmission electron microscopy (STEM) image. h, HAADF-STEM image for M-CoPc-400.

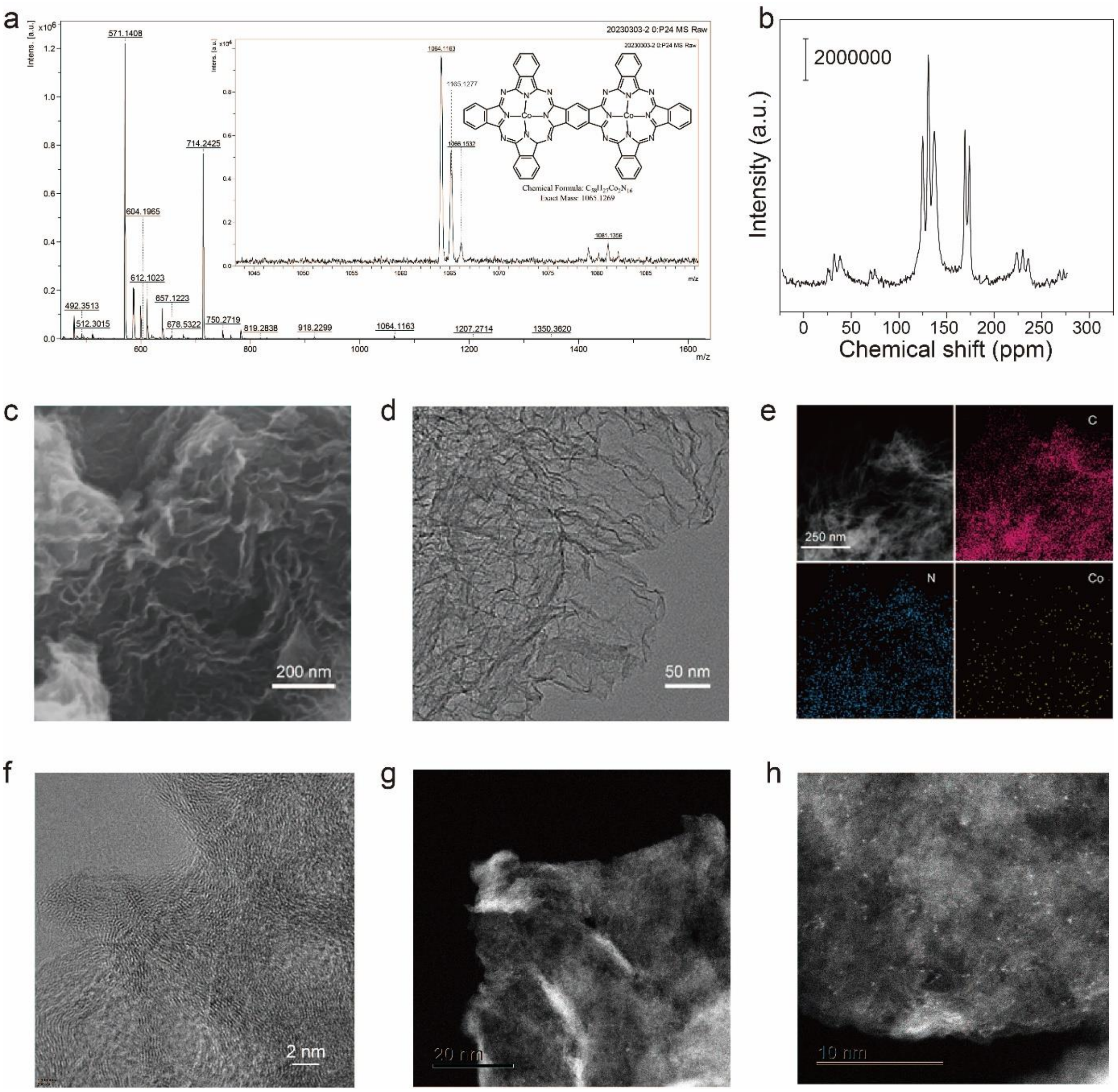

Figure S2 Morphology and structure characterizations for B-CoPc and B-CoPc-400. a, HR-MS and b, ^{13}C NMR spectra for B-CoPc. c, Scanning electron microscopy (SEM) image. d, Transmission electron microscopy (TEM) image. e, Energy dispersive X-ray (EDX) elemental mapping images. f, High-resolution TEM (HR-TEM) image. g, Scanning transmission electron microscopy (STEM) image. h, HAADF-STEM image for B-CoPc-400.

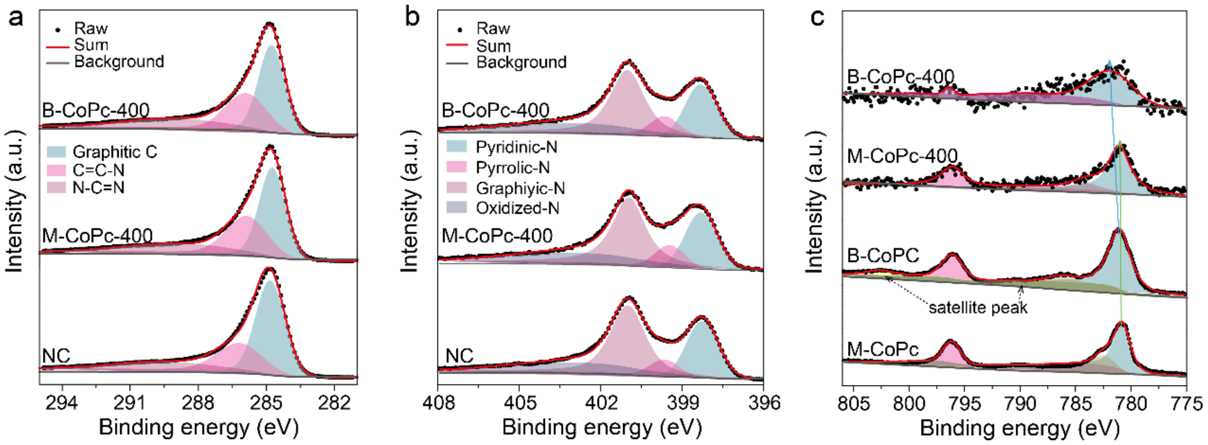

Figure S3 XPS spectra of M-CoPc-400 and B-CoPc-400: (a) C 1*s*, (b) N 1*s*, and (c) Co 2*p* XPS spectra. The C 1*s* XPS spectra can be deconvoluted into three peaks, corresponding to shake-up features, C=O, and sp^2 carbon, respectively. The N 1*s* XPS spectra can be deconvoluted into four peaks, which can be assigned to pyridinic nitrogen, pyrrolic nitrogen, graphitic nitrogen and oxidized nitrogen, respectively. The XPS results show that C and N in M-CoPc-400 and B-CoPc-400 have similar species. After heat treatment, Co 2*p* maintained a similar structure to M-CoPc and B-CoPc. The peak shape was slightly changed, indicating that M-CoPc and B-CoPc interacted with N-doped carbon.

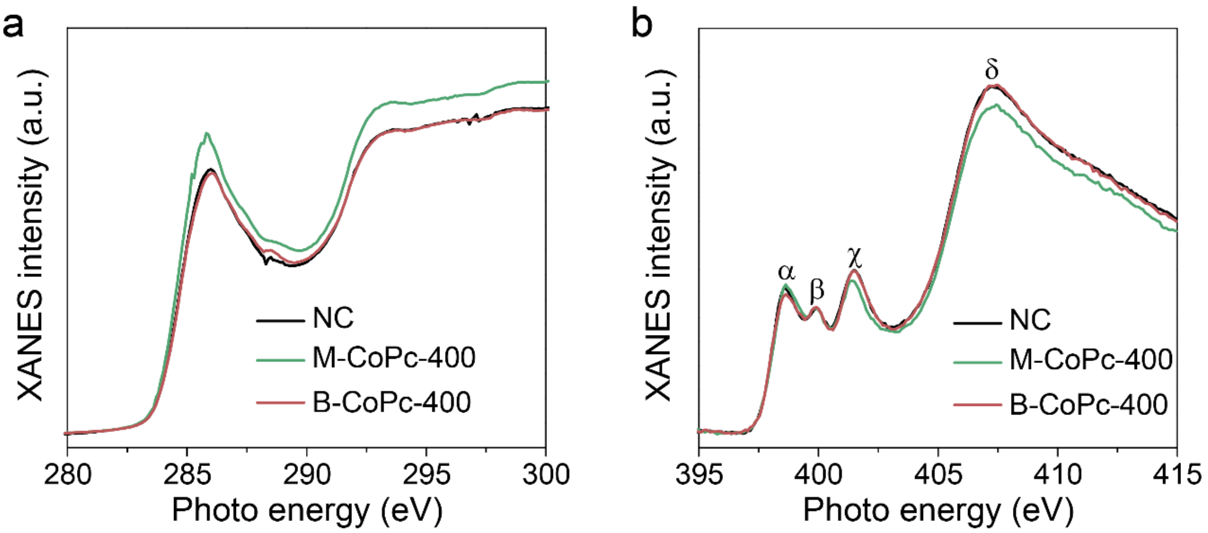

Figure S4 K-edge XANES spectra for C (a) and N (b) of NC, M-CoPc-400 and B-CoPc-400. One main feature is observed in the pre-edge region at ca. 285.6 eV (C-C). The C-C feature is related to electron transitions from C 1*s* orbital to π*(C=C) orbital, mostly coming from interlayered C=C bond configuration, in agreement with the results from XPS. In the N K-edge XANES spectra, both M-CoPc-400 and B-CoPc-400 display four features at 396.5 eV and 407.8 eV, associated with pyridinic nitrogen (α), pyrrolic nitrogen (β), graphitic nitrogen (γ) and oxidized nitrogen (δ), in line with N 1*s* XPS data.

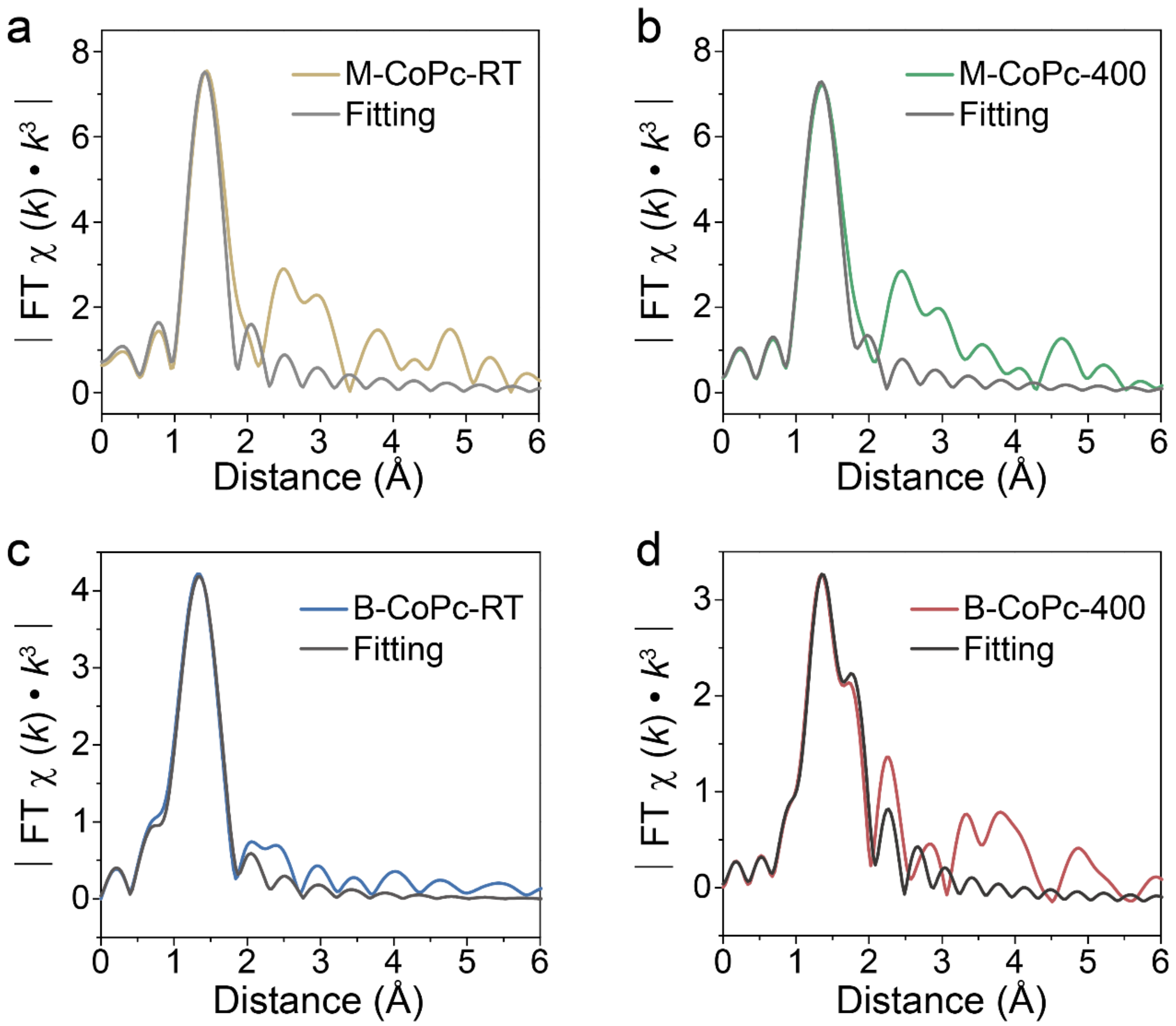

Figure S5 First-shell (Co-N) fitting of Fourier transformations of EXAFS spectra for M-CoPc-RT (a), M-CoPc-400 (b), B-CoPc-RT (c) and B-CoPc-400 (d). EXAFS spectra were fitted using the FEFF 6.0 code.

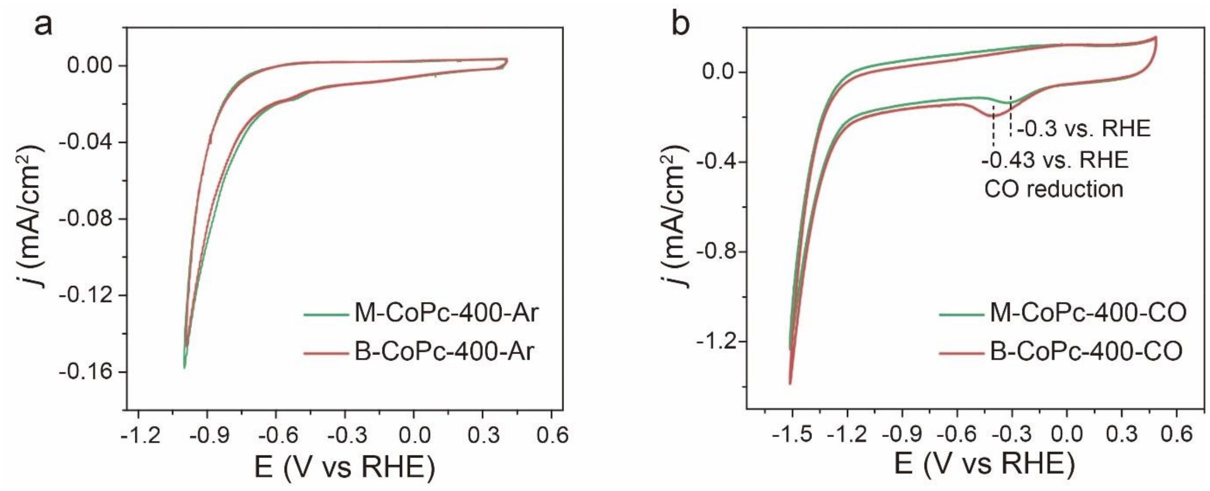

Figure S6 Cyclic voltammetry curves recorded in Ar-saturated (a) and CO-saturated (b) 0.5 M KOH aqueous solution.

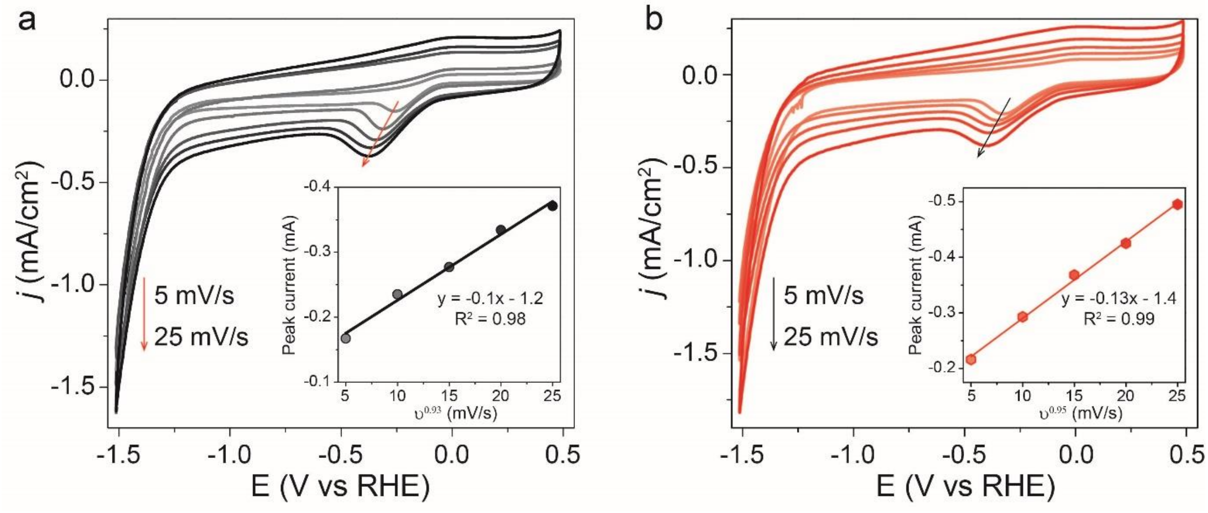

Figure S7 Cyclic voltammetry curves recorded at a scan rate of 5, 10, 15, 20, and 25 mV/s over (a) M-CoPc-400 and (b) B-CoPc-400 electrode in CO-saturated 0.5M KOH. Generally, the reduction peak current (I_p) obeys the power law relation with scan rate (ν): $I_p = a \times v^b$, where I_P is the peak current, a and b are adjustable parameters. The value of b can be estimated from the slope of the log (I_P) vs. log (ν) plot. There are two well-defined conditions: if b is close to 1, indicating purely surface reaction-controlled mechanism and b = 0.5, indicating purely diffusion-controlled mechanism. As revealed in the inset of Figure S9a and b, the b value for M-CoPc-400 and B-CoPc-400 are estimated to be ~0.93 and ~0.95, respectively.

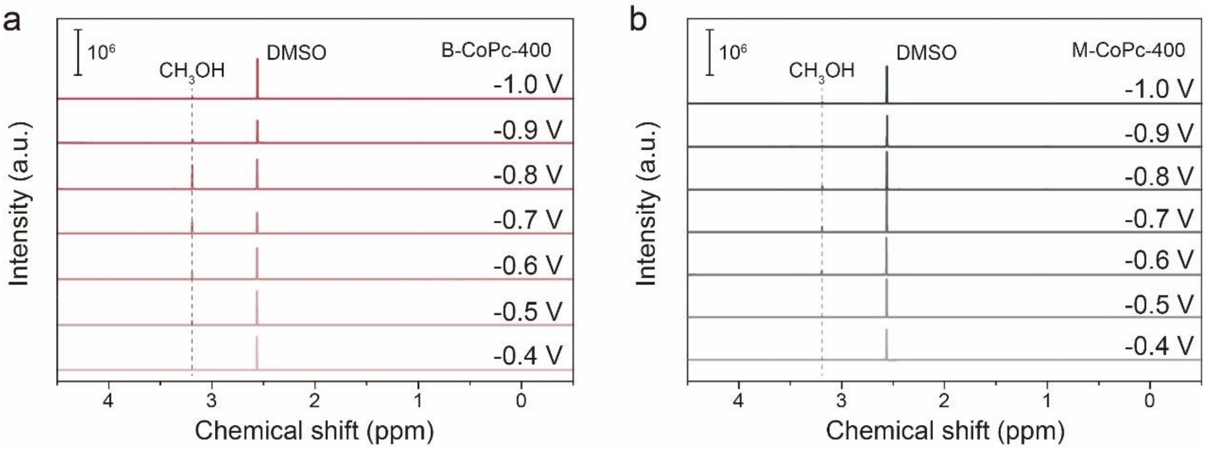

Figure S8 ^{1}H 1D NMR spectra of products and DMSO at different potentials (vs. RHE) for B-CoPc-400 (a) and M-CoPc-400 (b), respectively.

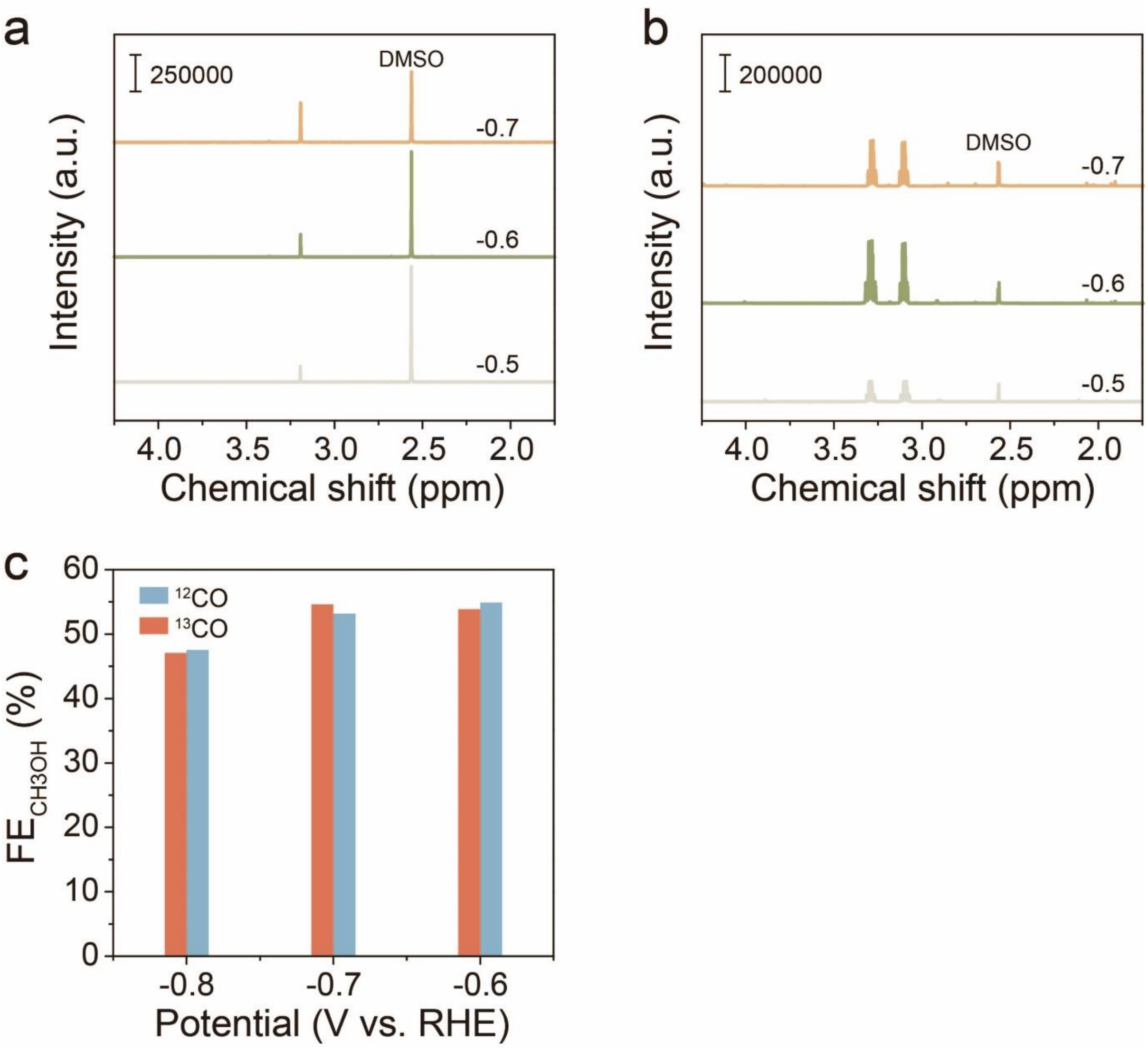

Figure S9 ^{1}H 1D NMR spectra of products and DMSO recorded at different potentials (vs. RHE) over M-CoPc-400 (a) and B-CoPc-400 (b). c, Comparison of Faradaic efficiency of CH_3OH with ^{13}CO and ^{12}CO as the reactant.

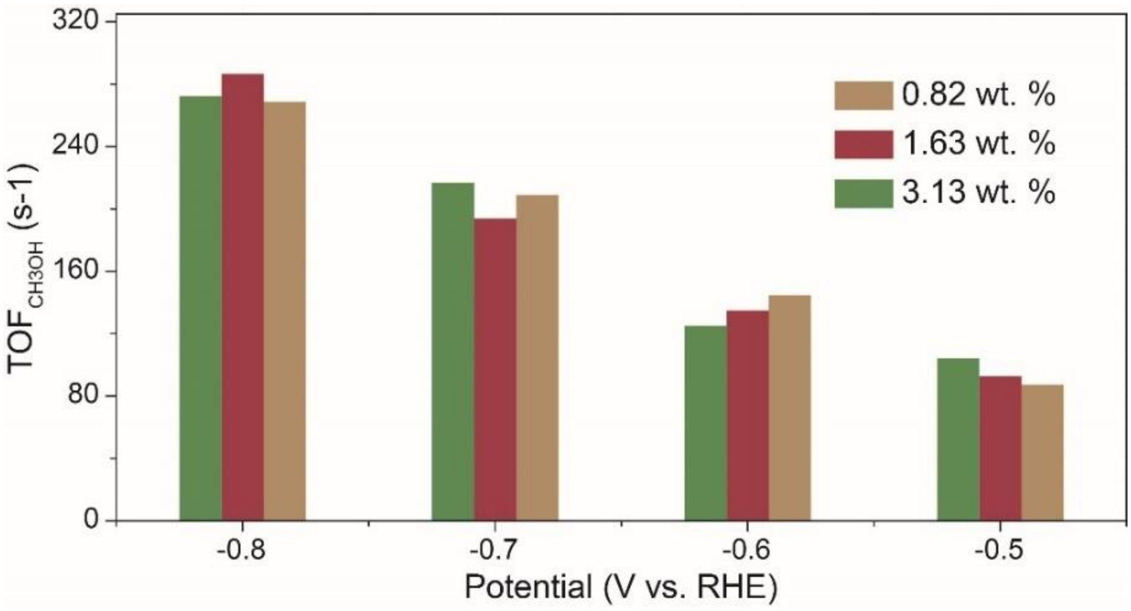

Figure S10 Potential-dependent product selectivity for CO electroreduction over B-CoPc-400 with different Co loading amount.

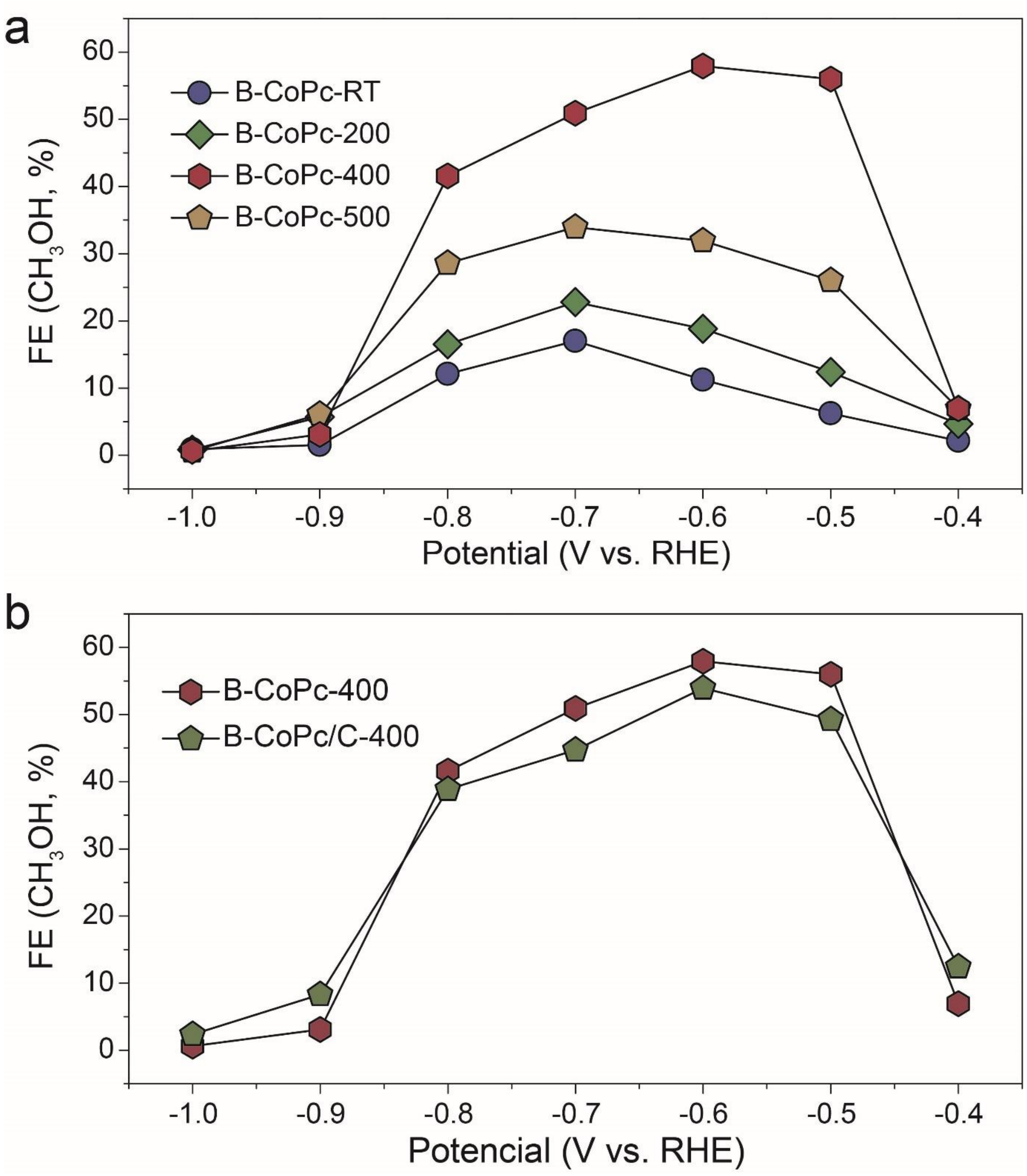

Figure S11 a, Potential-dependent product selectivity for CO electroreduction catalyzed by B-CoPc-X (X represents treatment temperature). b, Potential-dependent product selectivity for CO electroreduction catalyzed by B-CoPc-400 and B-CoPc/C-400 (B-CoPc/C-400 represents Vulcan carbon black as support to prepare the catalyst).

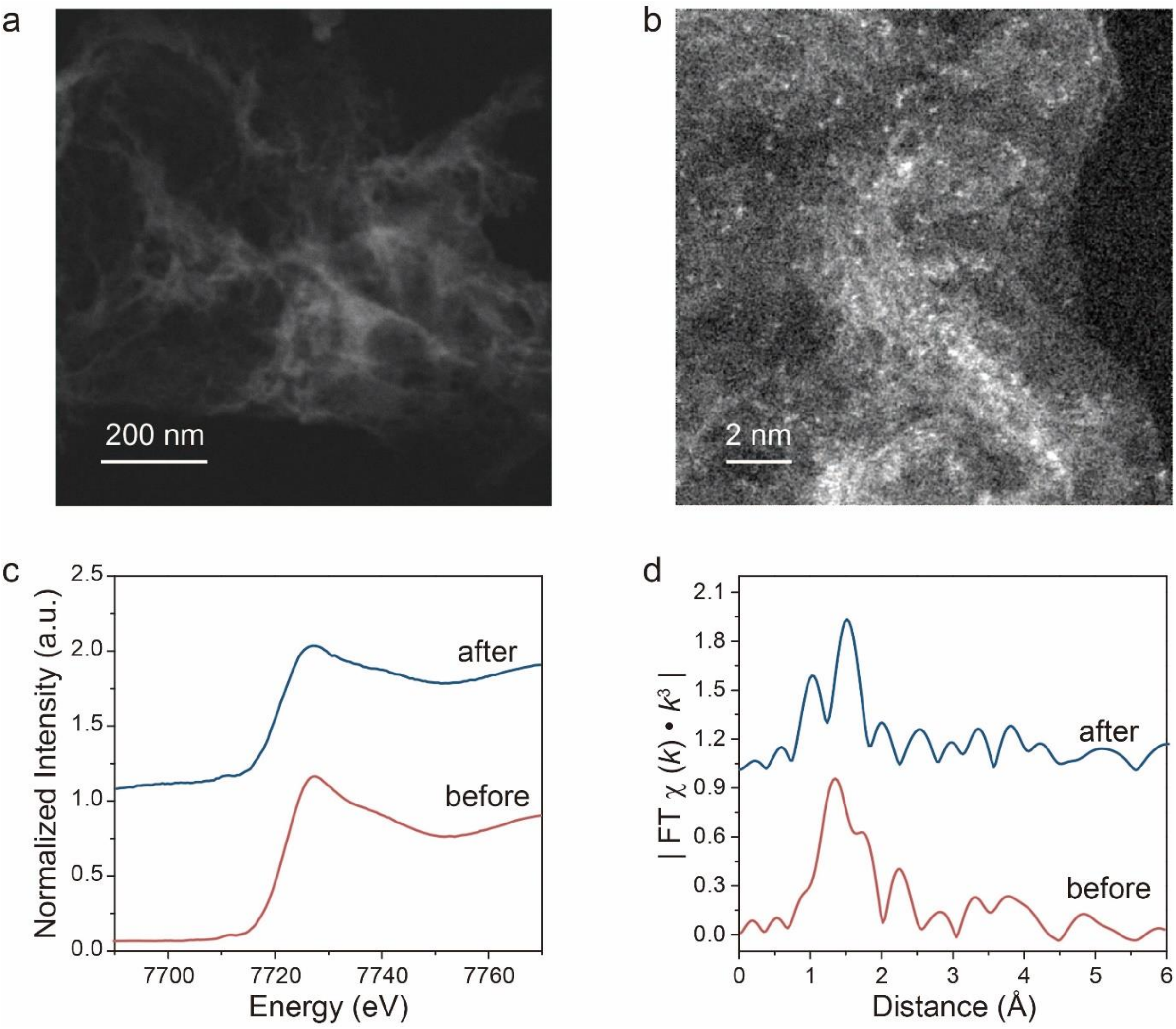

Figure S12 HRTEM (a) and HADDF-STEM (b) of B-CoPc-400 after CORR. c, Co K-edge XANES spectra for B-CoPc-400 before and after CORR. d, The corresponding Fourier transformation (FT)-EXAFS spectra.

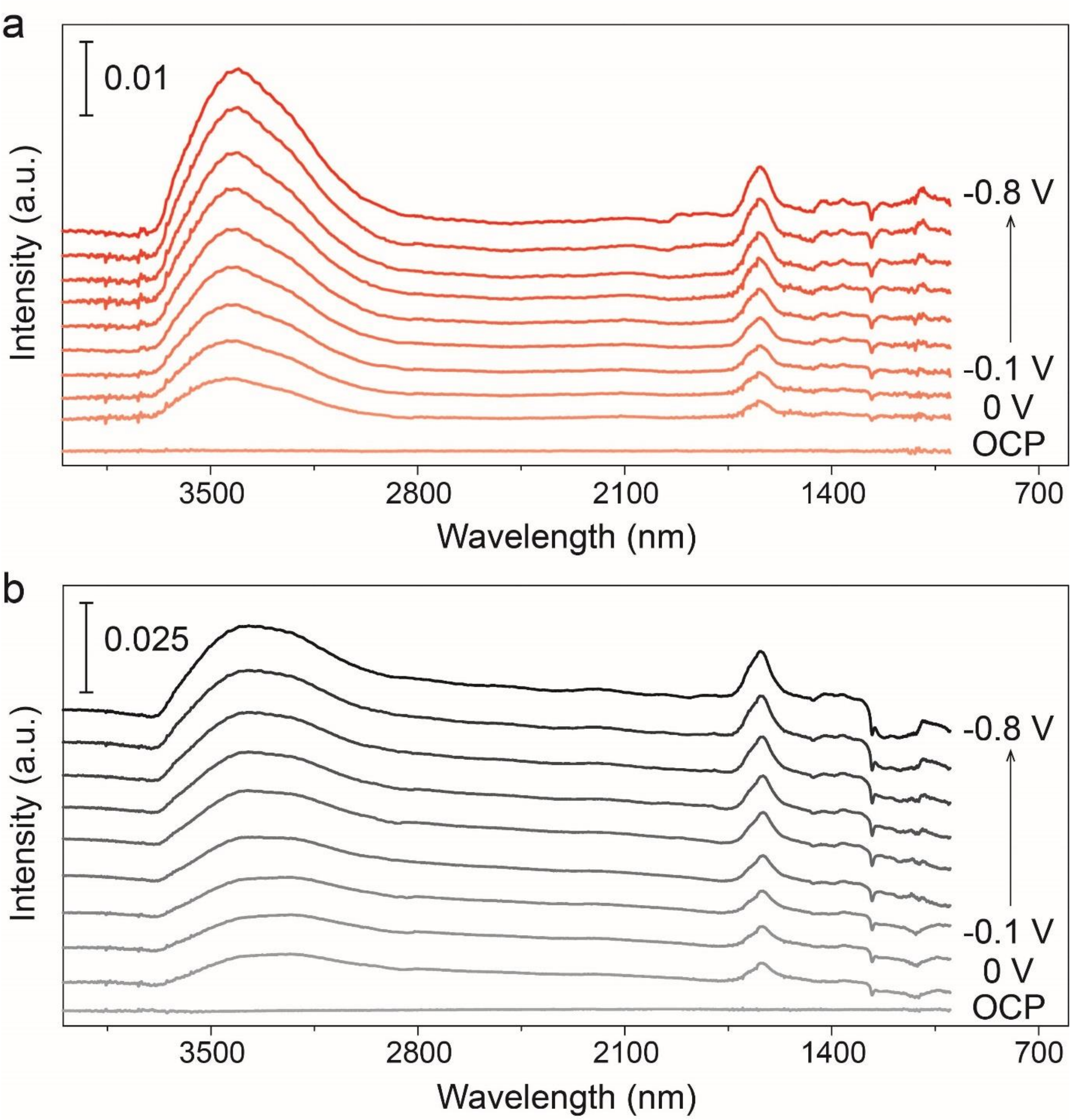

Figure S13 *Operando* ATR-SEIRAS spectra recorded on M-CoPc-400 (a) and B-CoPc-400 (b) in Ar-saturated 0.5 M KOH.

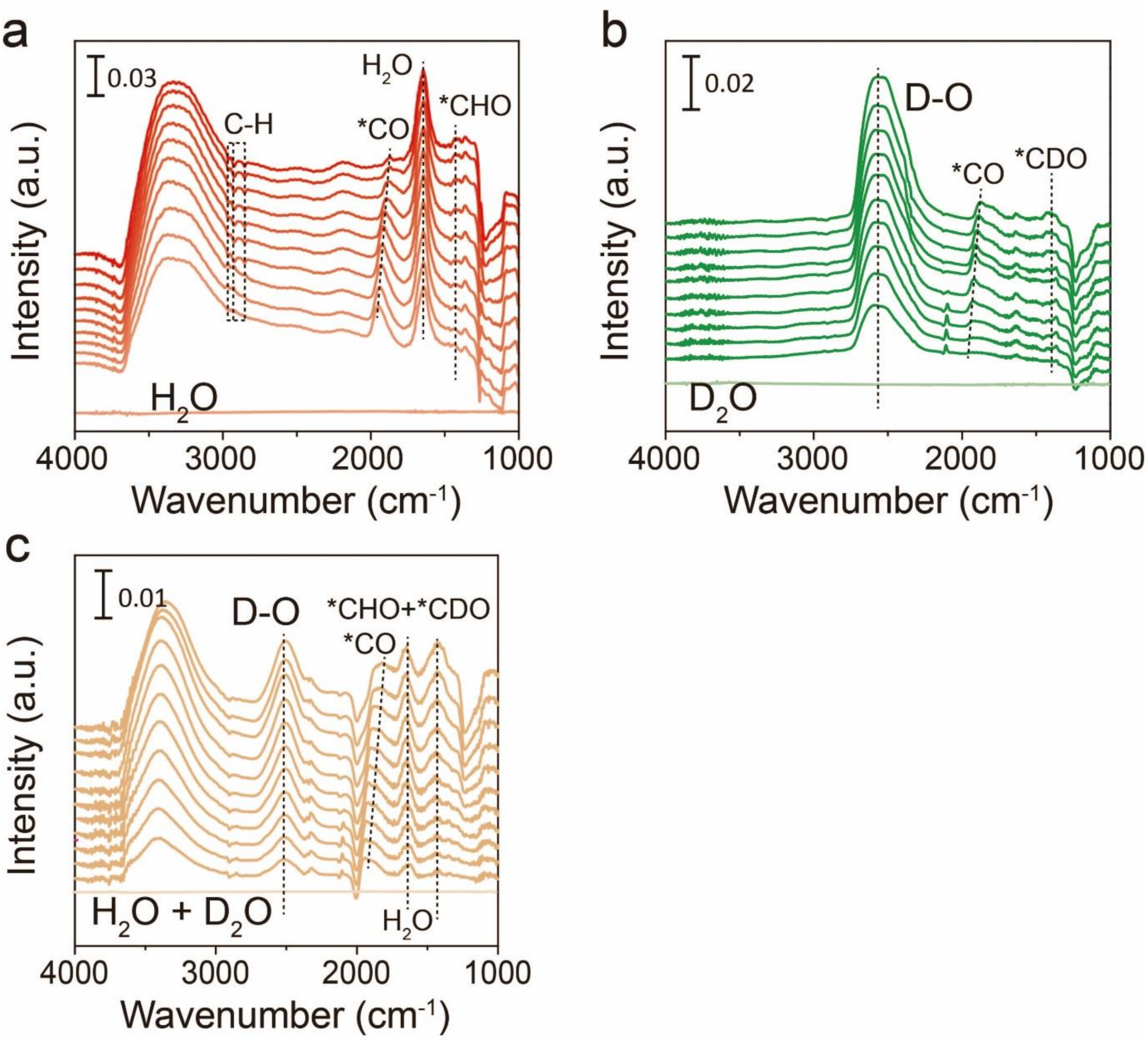

Figure S14 *Operando* ATR-SEIRAS spectra recorded over B-CoPc-400 in CO-saturated 0.5 M $KHCO_3$ with H_2O (a), D_2O (b), and H_2O + D_2O (c) as solvent.

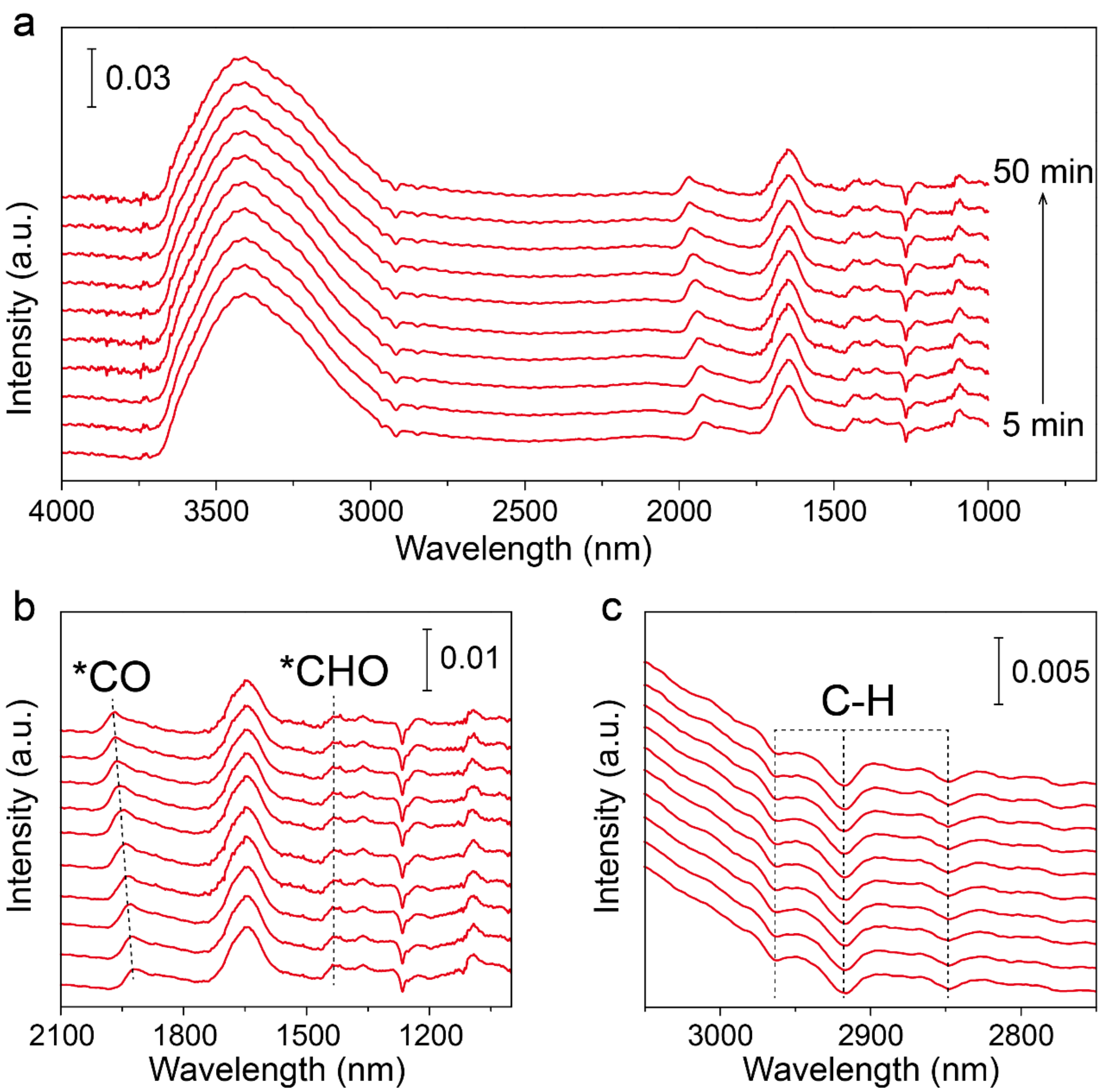

Figure S15 (a-c) Time-dependent *operando* ATR-SEIRAS spectra recorded on B-CoPc-400 in CO-saturated 0.5 M KOH.

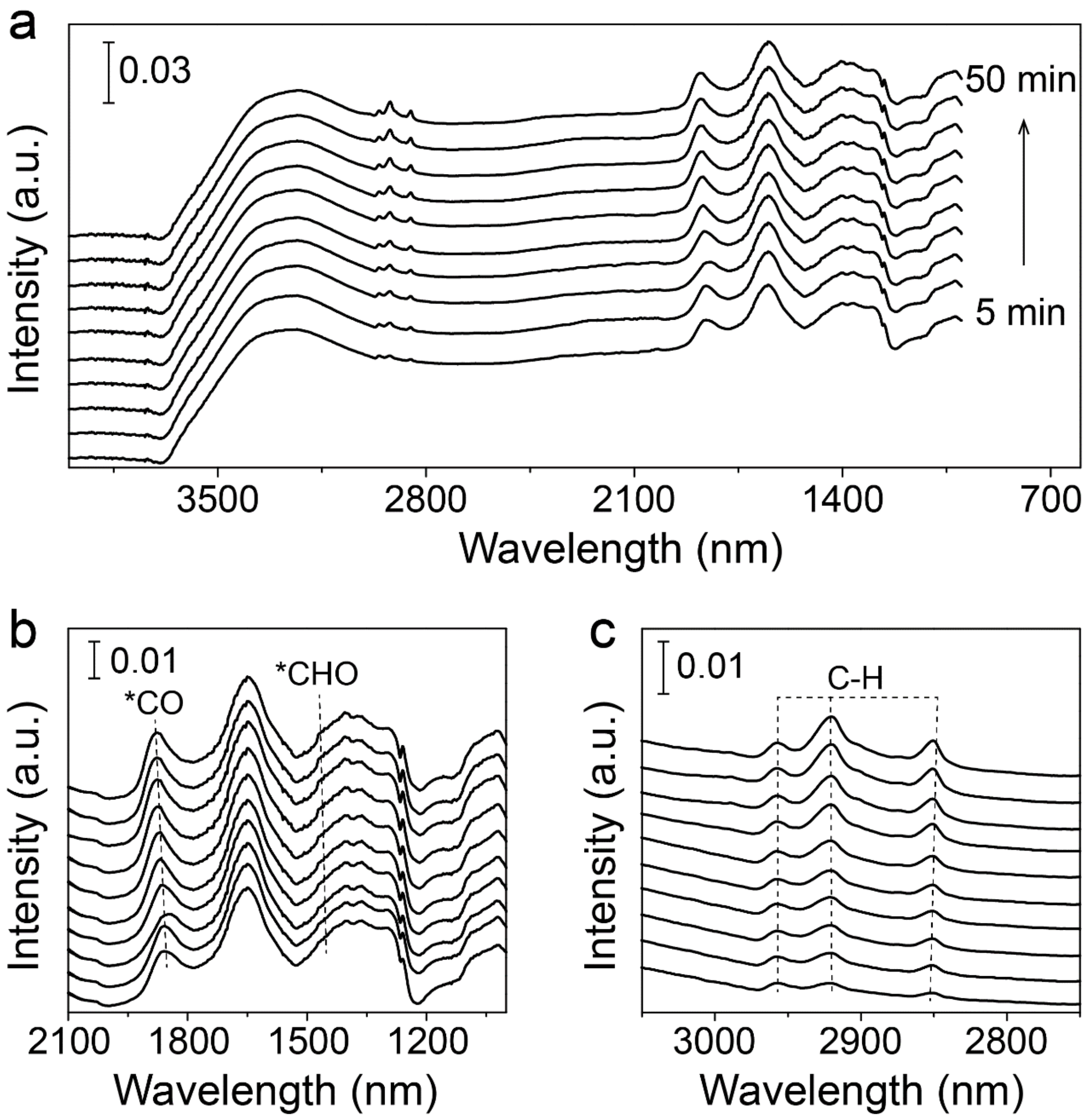

Figure S16 (a-c) Time-dependent *operando* ATR-SEIRAS spectra recorded on M-CoPc-400 in CO-saturated 0.5 M KOH.

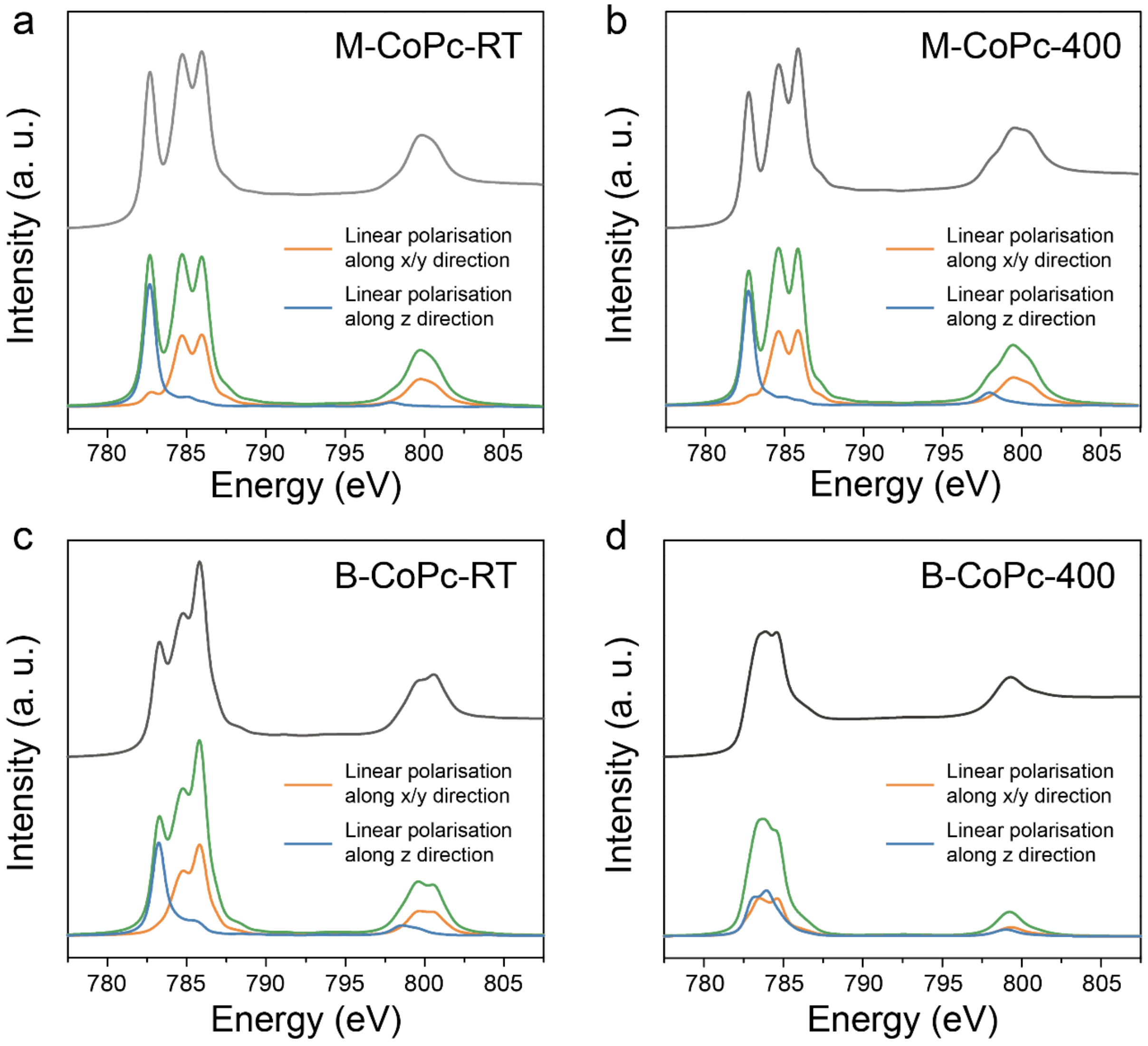

Figure S17 The simulated X-ray absorption spectra at Co $L_{2,3}$-edge of M-CoPc-RT (a), M-CoPc-RT-400 (b), B-CoPc-RT (c) and B-CoPc-RT-400 (d) at room temperature.

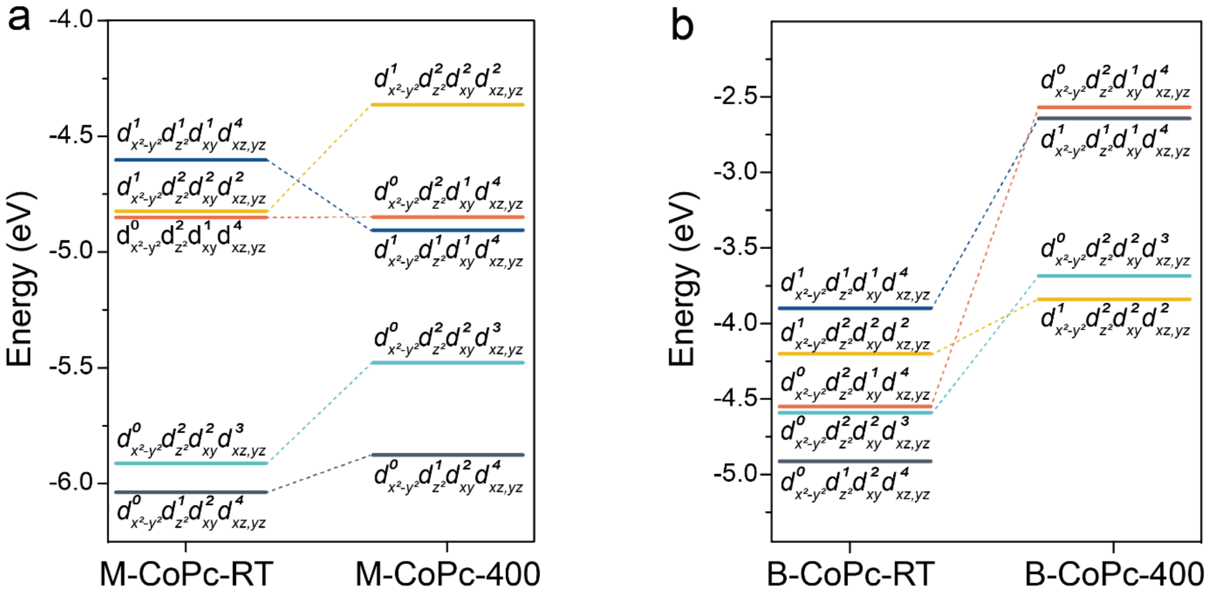

Figure S18 Energy of the cobalt center in different 3d electron configurations for (a) M-CoPc-RT and M-CoPc-400 as well as (b) B-CoPc-RT and B-CoPc-400.

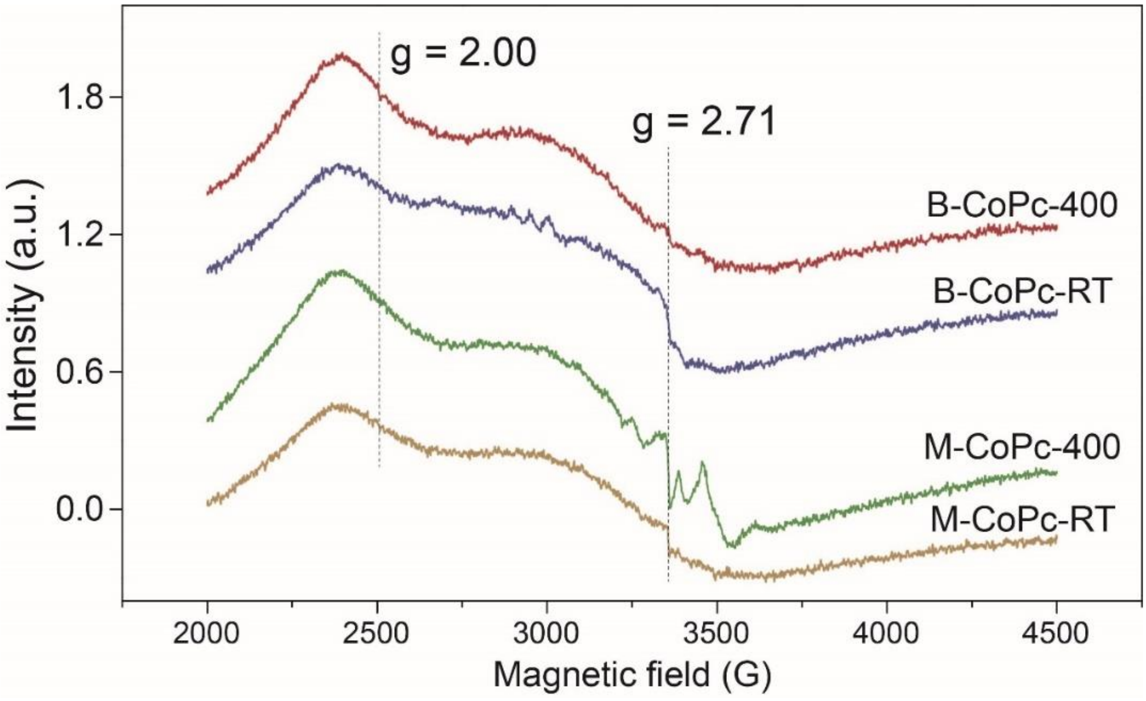

Figure 19 EPR spectra of various samples.

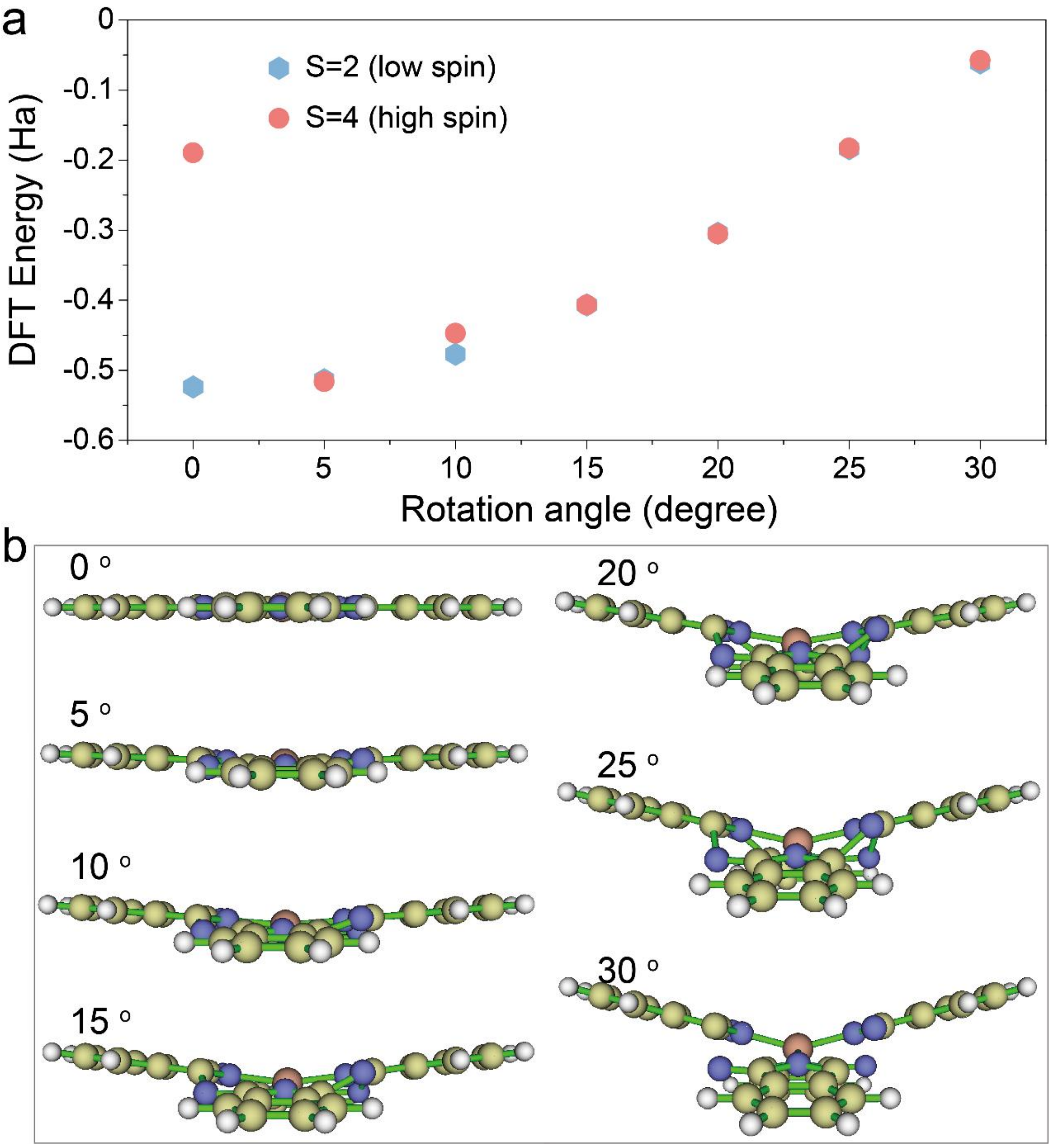

Figure S20 (a) The effect of structure distortion on spin state of Co^{2+} in CoPc. (b) The optimized structure of LS-Co and HS-Co with different rotation angles.

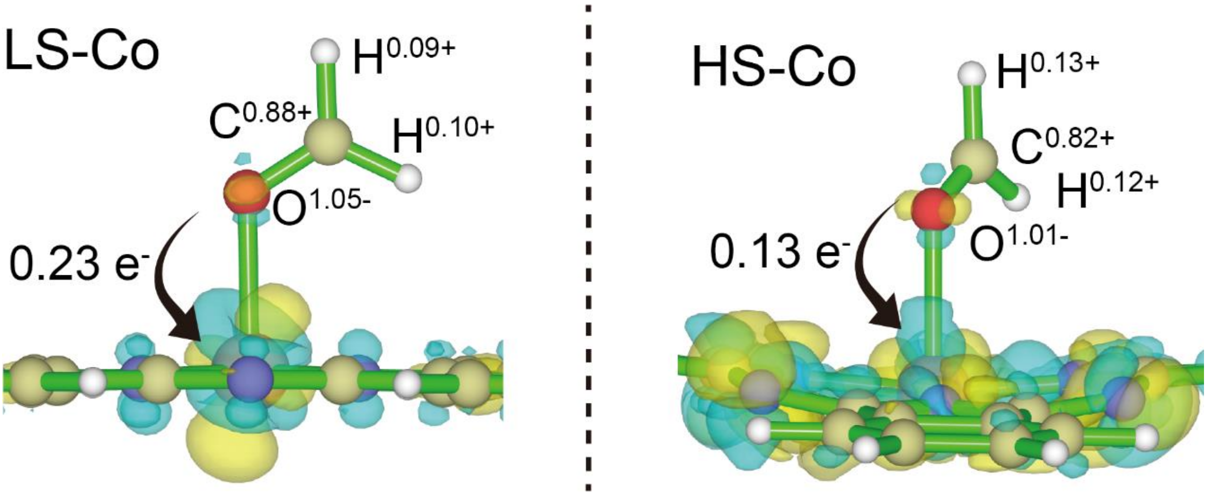

Figure S21 Schematic illustration showing the *CH_2O adsorption on Co center of LS-Co and HS-Co. Blue, yellow, pink, gray and white balls represent N, C, Co, O and H atoms, respectively, and the faint yellow and cyan regions refer to the increased and decreased charge density.

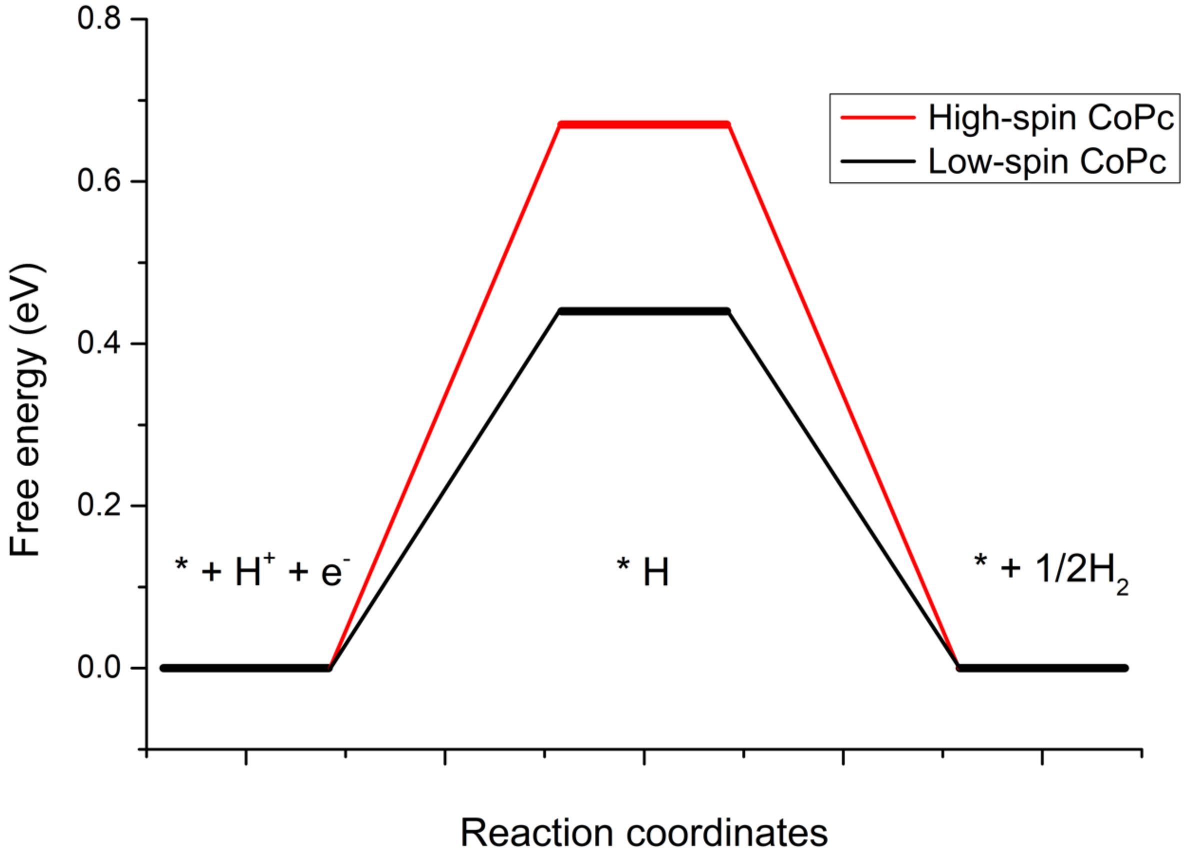

Figure S22 Potential energy profiles for HER over LS-Co and HS-Co.

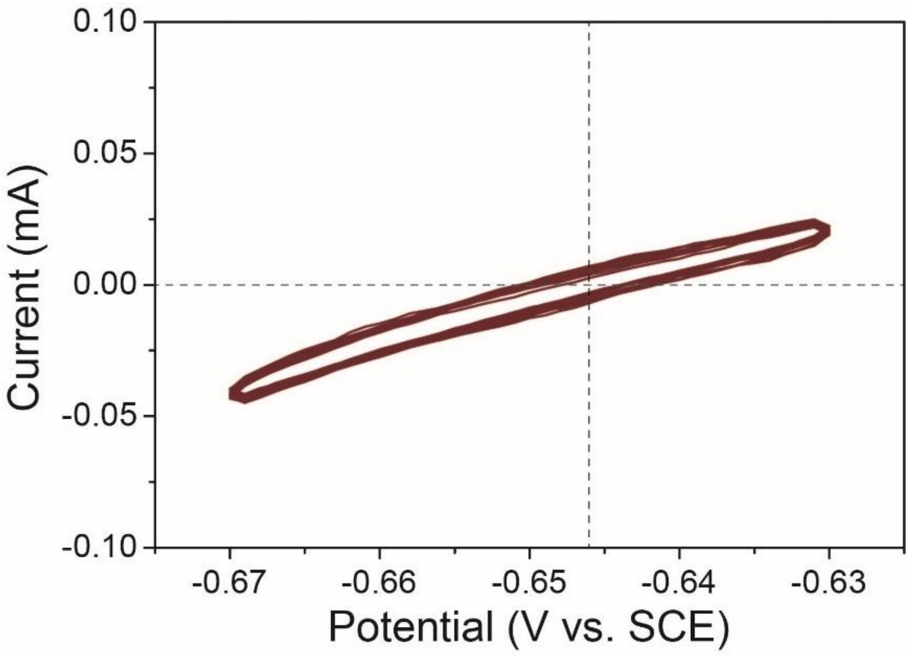

Figure S23 Cyclic voltammograms of Pt electrode at a scan rate of 1 mV/s in H_2 saturated 0.1 M KCl solutions.

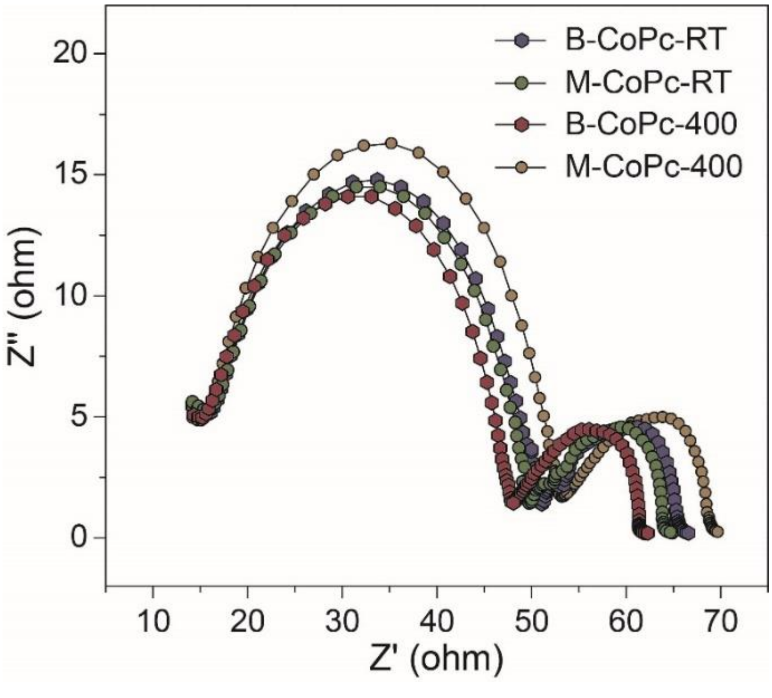

Figure S24 The EIS spectrum for M-CoPc-RT/400 and B-CoPc-RT/400.

Table S1. Structural parameters extracted from the Co K-edge EXAFS fitting. (S_0^2=0.76).

Sample	*Scattering pair*	*CN*	*R(Å)*	$\sigma^2(10^{-3}Å^2)$	$\Delta E_0(eV)$	*R factor* (10^{-2})
M-CoPc-RT	Co-N	4.2	1.89	3.0	3.4	6.7
M-CoPc-400	Co-N	4.7	1.87	3.0	-6.2	5.4
B-CoPc-RT	Co-N	4.7	1.92	8.0	-8.1	1.8
B-CoPc-400	Co-N	4.1	2.10	5.4	1.4	1.6
	Co-N	0.6	1.90	5.4	1.4	1.6

For the EXAFS fitting in Table S1, S_0^2 is the amplitude reduction factor; CN is the coordination number; *R* is interatomic distance (the bond length between Fe and Mo central atoms and surrounding coordination atoms); σ^2 is Debye-Waller factor (a measure of thermal and static disorder in absorber-scatterer distances); ΔE_0 is edge-energy shift (the difference between the zero kinetic energy value of the sample and that of the theoretical model). *R* factor is used to value the goodness of the fitting.

S_0^2 This value was fixed during EXAFS fitting, based on the known structure of Co foil.

Error bounds that characterize the structural parameters obtained by EXAFS spectroscopy were estimated as N ± 20%; *R* ± 1%; σ^2 ± 20%; ΔE_0 ± 20%.

Table S2. FE of all the products.

Catalysts	Potential (V vs. RHE)	FE(H_2)	FE(CH_3OH)	FE (total)
M-CoPc-400	-0.4	48%	5%	53%
	-0.5	74%	11%	85%
	-0.6	78%	13%	91%
	-0.7	75%	19%	94%
	-0.8	80%	16%	96%
	-0.9	92%	1%	93%
	-1.0	93%	2%	95%
B-CoPc-400	-0.4	35%	8%	43%
	-0.5	43%	44%	87%
	-0.6	33%	57%	90%
	-0.7	38%	56%	94%
	-0.8	44%	48%	92%
	-0.9	90%	4%	94%
	-1.0	92%	2%	94%

Table S3. Comparison of CORR performance between our prepared catalyst and those reported in the literature.

Catalyst	Current density (mA)	$FE_{methanol}$ (%)	Reference
B-CoPc-400	154	57	**This work**
CoPc	4.77	14.3	*Angew. Chem. Int. Ed.* 2019, 58, 16172
CoPc/MWCNT	3.8	14	*Chem. Eur. J.* 2022, 28, e202200697
